# Assessing the Significance and Progression of Too Small to Characterize Lesions in Hepatic CT Scans of Patients With a Cancer History: A Two‐Center Cross‐Sectional Study

**DOI:** 10.1002/hsr2.70374

**Published:** 2025-03-02

**Authors:** Farzaneh Khoroushi, Mohammad Moeini, Lida Jarahi, Ehsan Hassannejad, Farnaz Kharghani, AmirAli Moodi Ghalibaf

**Affiliations:** ^1^ Faculty of Medicine Mashhad University of Medical Sciences Mashhad Iran; ^2^ Ghaem Hospital, Faculty of Medicine Mashhad University of Medical Sciences Mashhad Iran; ^3^ Community Medicine Department, Faculty of Medicine Mashhad University of Medical Sciences Mashhad Iran; ^4^ Department of Radiology, School of Medicine Birjand University of Medical Sciences Birjand Iran; ^5^ Department of Radiology, School of Medicine Mashhad University of Medical Sciences Mashhad Iran; ^6^ Student Research Committee Birjand University of Medical Sciences Birjand Iran

**Keywords:** cancer, computed tomography, liver, too small to characterize

## Abstract

**Background and Aim:**

During staging computed tomography (CT) of patients with cancer, lesions that are too small to characterize (TSTC) on the liver are frequently observed. Considering the significance of these lesions, this study aimed to evaluate the relevance and progression of TSTC lesions on hepatic CT scans in patients with a history of cancer.

**Method:**

This cross‐sectional study was carried out on 50 patients with a history of cancer who underwent contrast‐enhanced abdominal CT scans. These patients were referred to Ghaem Hospital and Imam Reza Hospital, Mashhad, between March 2022 and 2023. In this study, demographic and clinical information were documented. The CT scans of all patients included in the study were evaluated by two experienced radiologists who assessed the number and size of TSTC lesions. Next, each patient was called to perform a follow‐up CT scan 6 months later. Then, the size and number of TSTC lesions were compared with the values recorded in the previous round, and their progress was evaluated.

**Results:**

The study included 50 patients, of which 46% were male and 54% were female. The mean patients' age was 54.88 ± 13.60 years. In the following CT scan, among the patients with TSTC lesions, nine (18%) had liver metastases, while 41 (82%) did not. There was no significant difference between the groups with and without metastases in terms of the number of TSTC lesions (*p* = 0.051), the size of the largest TSTC lesion (*p* = 0.960), and the duration of chemotherapy (*p* = 0.330). However, the history of chemotherapy was statistically significant between the two groups (*p* < 0.001).

**Conclusion:**

The incidence of metastasis in individuals with TSTC lesions was 18%, higher than that reported in similar studies. The identification of these lesions is crucial for accurate diagnosis and treatment planning.

## 1 Introduction

Computed tomography (CT) scans play a vital role in the identification of hepatic metastases. In unenhanced CT scans, these metastases usually appear as hypoattenuating and exhibit less enhancement compared to the surrounding liver tissue after contrast administration [1]. The triple‐phase hepatic CT protocol proves to be an effective method for evaluating focal hepatic lesions, hypervascular hepatic metastases, and endocrine tumors. This protocol involves acquiring images in the late arterial, portal venous, and delayed phases [2].

The identification and characterization of hepatic lesions through CT scans are primarily determined by dissimilarities between the lesion and background, along with enhancement kinetics [3]. When viewed on portal vein phase imaging, metastases are often characterized by low‐attenuation masses and a hypovascular nature in comparison to hepatic background tissue. Despite the frequent irregularity of the margins, they can occasionally be well‐defined [4]. The presence of a hypoattenuating halo encircling the edges of a lesion is a strong predictor of malignancy. The main focus of staging CT scans in patients with suspected or confirmed malignancies is the early detection of hepatic metastases [5]. Distinguishing metastases larger than 15 mm in diameter from benign lesions of equivalent size is typically possible. However, this differentiation is increasingly challenging for smaller lesions [6].

The term “Too Small to Characterize” (TSTC) is employed in the field of radiology to indicate lesions of such small size that they cannot be precisely characterized through imaging scans. This frequently occurs due to problems associated with volume averaging during attenuation evaluation [7]. When it comes to hepatic lesions, TSTC lesions refer to those with a lesion size that is smaller than double the thickness of the reconstructed slice. The identification of these lesions presents a diagnostic challenge, necessitating accurate categorization by radiologists based on their clinical significance [8].

Several studies have investigated the importance and frequency of TSTC on hepatic CT scans, especially among patients with cancer. The studies have demonstrated that hepatic lesions of this nature are present in 12.7%–50% of cancer patients [9, 10, 11]. Although the majority of these lesions, which are coincidentally observed in patients with malignancies, are benign, a range of 5%–27.5% are ultimately determined to be malignant [12]. These statistics highlight the importance of accurate diagnosis of these small lesions. Nonetheless, the acquisition of a histological diagnosis for small lesions through biopsy is invasive, technically demanding, and often unattainable [10]. Considering the significance of these lesions, this study aimed to evaluate the relevance and progression of TSTC lesions on hepatic CT scans in patients with a history of cancer.

## 2 Material and Method

### 2.1 Study Protocol

In this study, demographic information, including age and gender, was recorded for each patient. Clinical information such as the patient's history of chemotherapy and duration of chemotherapy were also documented. Two experienced radiologists evaluated the CT scans of all patients included in the study. They assessed the number and size of TSTC lesions. The number of lesions and the diameter of the largest lesion (in millimeters) for each patient were documented on a checklist. Next, each patient was called to perform a follow‐up CT scan 6 months later. This time, the CT scan of the patients was evaluated by the same two radiologists, and the size and number of TSTC lesions were recorded on the checklist. Then, these items were compared with the values recorded in the previous round, and their progress was evaluated.

With the aim of reducing potential errors in all measurements, the values reported by both radiologists were recorded and compared. In the case of a discrepancy, the average value was used.

### 2.2 Study Population

This cross‐sectional study was conducted on 50 patients with a history of cancer who underwent abdominal CT scans with contrast and were referred to Ghaem Hospital and Imam Reza Hospital, Mashhad, Iran, between March 2022 and March 2023 by census sampling method. This study included patients who had a confirmed history of any malignancy, as determined by surgery, imaging, biopsy, or pathology. Additionally, these patients had at least one TSTC lesion smaller than 10 mm on hepatic CT scans and provided informed consent to take part in the research project. However, patients who were at risk of contrast nephropathy were not included in the study.

### 2.3 Ethical Considerations

The research project was thoroughly reviewed and approved by the Ethics Committee of Mashhad University of Medical Sciences (Approval No: IR.MUMS.MEDICAL.REC.1401.107). Prior to participating in the study, all patients provided informed consent, and their data were entered anonymously to ensure confidentiality.

### 2.4 Statistical Analysis

Descriptive data were characterized using means, standard deviations, and percentages. Before analyzing the data, the normality was assessed using the Shapiro–Wilk test. The Chi‐Square test was used to compare qualitative data. For comparing quantitative data between two groups, the independent sample T‐test was used in cases of normal distribution. Otherwise, the Mann–Whitney test was employed. The data was analyzed using a statistical software package (version 22, based in Chicago, IL, USA). A *p*‐value of less than 0.05 was deemed to indicate statistical significance.

## 3 Results

This study included 50 patients, 23 (46%) male and 27 (54.5%) female. The mean ± SD age of the patients was 54.88 ± 13.60 years. In the following CT scan, among the patients with TSTC lesions, nine (18%) had liver metastases, while 41 (82%) did not. Of the nine patients who had metastases, three patients had multiple lesions, and in six patients, the lesions had increased to more than 10 mm.

The clinical information of the patients, divided into groups with and without metastases, is presented in Table 1. According to the statistical analysis, there was no significant difference between the two groups (those with metastases and those without) in terms of the number of TSTC lesions (*p* = 0.051), size of the largest TSTC lesion (*p* = 0.960), and duration of chemotherapy (*p* = 0.330) in patients.

**Table 1:** The clinical information of the patients based on with and without metastases.

Variable	Metastatic group			Non‐metastatic group			*p*‐value
	Median	Minimum	Maximum	Median	Minimum	Maximum	
Number of TSTC lesion	2	1.5	3.5	2	1	3	0.051*
TSTC lesion size (mm)	9	6	10	7	4	8	0.960*
The duration of chemotherapy (hour)	16	16	21	18	16	24	0.330*

Abbreviation: TSTC, too small to characterize.

* Mann–Whitney test.

In the group of patients without metastasis, only 12 (29.26%) had a history of chemotherapy. However, all nine patients with metastasis had a history of chemotherapy. This difference was statistically significant (*p* < 0.001). Among cancers whose TSTC lesions had metastasized, breast cancer, renal cell carcinoma, and colon cancer had two cases each, and esophageal, stomach, and pancreatic cancers had one case each.

## 4 Discussion

The management protocol for TSTC in hepatic CT scans for early‐stage cancer is currently undefined. In comparison to CT scans, the inclusion of diffusion‐weighted and contrast‐enhanced imaging in magnetic resonance imaging (MRI) scans offers a more precise characterization of hepatic lesions [13]. In this study, we examined TSTC lesions in the hepatic CT scans of patients with a cancer history. The findings of this study revealed that 18% of individuals with TSTC lesions had metastases. The metastatic and non‐metastatic groups showed no significant differences in the number of TSTC lesions, size of these lesions, or duration of chemotherapy. The only significant difference between the two groups was the history of chemotherapy. Specifically, 29.26% of patients without metastasis had undergone chemotherapy, while all nine patients with metastasis had a history of chemotherapy.

Van den Broek et al. determined that 54% of TSTC cases occurred in colorectal cancer, and patients diagnosed with early‐stage rectal cancer frequently exhibited lesions in the hepatic region. On average, three hepatic lesions were observed in these patients [11]. A restricted number of studies have investigated uncertain liver lesions in patients diagnosed with colorectal cancer. The incidence of these indeterminate hepatic lesions reported in these studies ranges from 17% to 30% [14, 15, 16]. In their study, Khalil et al. found that 29.4% of women diagnosed with breast cancer, who initially showed no signs of hepatic metastasis on their CT scan, were later found to have at least one hepatic lesion identified as TSTC [9]. In a study conducted by Foley and Hamilton, it was discovered that among 289 patients diagnosed with rectal cancer, 22 patients (7.6%) displayed TSTC hepatic lesions as detected on CT scans. Among these lesions, 12 were categorized as simple cysts, while five were determined to be benign (mostly haemangiomas), and another five were confirmed as metastases [17]. The occurrence of lesions in the current study was notably higher than that reported in previous studies. Additionally, as we followed up with new cases, it was found that the frequency and/or the size of the lesions increased significantly which can be defined as metastasis due to the Response Evaluation Criteria in Solid Tumors (RECIST) criteria; this can be a reason for the rate of 18% of TSTC metastasis in our study; which is a little bit lower than some previous studies that defined it as approximately 20% [17]. Moreover, this can be attributed to the possibility of submillimeter lesions being overlooked during a single review. The CT scans in this investigation were evaluated by two radiologists, which could have contributed to the higher detection rate. Over the past few years, there have been notable improvements in the detection of small liver lesions through CT scans, thanks to advancements in the spatial and contrast resolution capabilities of CT scanners. The ongoing development of technology and the implementation of computer‐assisted diagnostic systems are expected to sustain this progression.

According to a study conducted by van den Broek et al., a total of 122 hepatic lesions were detected on the CT scans of patients diagnosed with colorectal cancer. Among them, 95 (78%) were categorized as indeterminate. Moreover, 20% (75 lesions) were determined to be cysts, while hemangiomas accounted for 2% (two cases) [11]. Patterson et al. conducted another study wherein they identified 22 lesions as benign in patients with breast cancer, 11 were indeterminate, and five were metastatic. In the study above, only 5% of the TSTC lesions were diagnosed as malignant [18]; our study found that 18% of the TSTC cases were metastatic. These TSTC cases were observed in patients with various types of cancers, including breast cancer, renal cell carcinoma, colon cancer, esophageal cancer, stomach cancer, and pancreatic cancer.

Khalil et al. carried out research on TSTC in patients who had breast cancer. Their study demonstrated that in between 92.7% and 96.9% of female patients diagnosed with breast cancer, the TSTC lesions in the liver were not due to metastasis [9]. Littler et al. conducted a study in which they found that out of 289 patients with colorectal cancer, 22 (7.6%) had TSTC hepatic lesions on CT scans. The mean lesion size was 11 mm. Out of these, twelve were recognized as simple cysts, five were identified as benign primary hemangiomas, and five were classified as metastases. According to their proposal, while simple cysts can be diagnosed with confidence and metastases can be detected with high sensitivity, there is still a lack of certainty in the diagnosis. Most hemangiomas cannot be definitively diagnosed due to their apparent diffusion restriction, which is indicative of sluggish vascular flow [19]. The current study's findings indicate no significant difference in the size of the TSTC lesions between the metastatic and non‐metastatic groups. However, the two groups observed a significant difference in the history of chemotherapy. All patients with metastases had a history of chemotherapy. However, due to the limited sample size, it was impossible to draw broad conclusions in this area. Therefore, conducting further studies with a larger sample size and in a multicenter setting in this field would be beneficial.

The findings from earlier studies on indeterminate hepatic lesions in patients with colorectal cancer reveal that a substantial number of these uncertain hepatic lesions are benign [20]. However, certain studies suggest that a small percentage of indeterminate tiny liver lesions reveal evidence of metastatic disease in follow‐up imaging. As a result, these studies suggest the need for continuous imaging monitoring in patients with colorectal cancer who have indeterminate hepatic lesions on CT scans [21, 22]. The researchers suggested that it is advisable to forgo additional hepatic imaging when indeterminate hepatic lesions are identified in cancer patients who are deemed relatively low‐risk [23]. The results of the present study suggest that there is no statistically significant difference in the number and size of TSTC lesions between the metastatic and non‐metastatic groups, and as a result, these variables do not hold predictive value for lesion progression.

TSTC lesions are frequently identified through medical imaging techniques like CT or MRI scans. Nevertheless, the diminutive size of these entities hinders the identification of their distinct features. The identification of these lesions is essential for precise diagnosis and treatment planning. Follow‐up imaging studies or other diagnostic tests may be necessary to observe changes in lesion size or appearance over time. This can assist in determining its nature and provide information for subsequent medical decisions. When it comes to hepatic lesions, TSTC lesions often necessitate additional imaging, biopsy, or clinical observation in order to determine their benign or malignant nature [24].

Additionally, our findings indicated that chemotherapy duration is not significantly associated with liver metastasis. However, due to the previous studies, the chemotherapy duration can potentially be associated with liver metastasis which can be related to the tumor biology, patient response to treatment, liver function, and overall treatment goals, despite this relationship being an area of controversy [25, 26, 27]. It should be noted that patients with compromised liver function may also experience altered pharmacokinetics, potentially leading to increased toxicity or reduced efficacy of chemotherapy, which could contribute to the development of new metastases [28]. According to the current knowledge, there is non‐certain data to determine the exact relationship between chemotherapy duration and liver metastasis, which needs further investigations.

## 5 Limitations and Suggestions

Few studies have evaluated the relationship between hepatic TSTC lesions and cancerous diseases. In the present context, our study offered radiologists with clinically relevant insights into TSTC. The primary limitation of the current study is the small sample size. Also inclusion of diffusion‐weighted and contrast‐enhanced MRI in future studies can offer a better characterization of hepatic lesions. Nevertheless, the present study involved a greater number of metastatic cases, thereby permitting a thorough comparison between metastatic and non‐metastatic cases. Notwithstanding these findings, it would be advantageous to carry out additional studies in a multicenter setting with larger sample sizes. This enabled the results to be generalized more effectively.

## 6 Conclusions

The assessment of TSTC lesions in hepatic CT scans of patients with a cancer history reveals no disparity in the size and number of TSTC lesions between the metastatic and non‐metastatic groups. However, it was found that all patients with metastasis had a history of chemotherapy. Therefore, it is essential to identify these lesions in order to achieve accurate diagnosis and effective treatment planning. Further studies should be conducted to supplement the current study's findings in this field.

## Author Contributions

**Farzaneh Khoroushi:** conceptualization, investigation, project administration, data curation, supervision, writing–original draft. **Mohammad Moeini:** supervision, data curation, project administration, conceptualization, investigation. **Lida Jarahi:** methodology, software, formal analysis. **Ehsan Hassannejad:** data curation, writing–original draft, writing–review and editing, methodology, formal analysis. **Farnaz Kharghani:** data curation, writing–review and editing, writing–original draft, methodology, formal analysis. **AmirAli Moodi Ghalibaf:** writing–review and editing, writing–original draft.

## Ethics Statement

The method was approved in compliance with scientific and ethical standards. All methods were performed in line with the relevant guidelines and regulations. The Medical Ethics Committee of Mashhad University of Medical Science approved this study.

## Consent

All patients were informed and signed the informed consent.

## Conflicts of Interest

The authors declare no conflicts of interest.

## Transparency Statement

The lead author Farnaz Kharghani affirms that this manuscript is an honest, accurate, and transparent account of the study being reported; that no important aspects of the study have been omitted; and that any discrepancies from the study as planned (and, if relevant, registered) have been explained.

## Data Availability

The data that support the findings of this study are available on request from the corresponding author. The data are not publicly available due to privacy or ethical restrictions. The datasets created during the current study are not publicly accessible due to the possibility of compromising the privacy of individuals.
